# Molecular Functions of Long Non-Coding RNAs in Plants

**DOI:** 10.3390/genes3010176

**Published:** 2012-03-08

**Authors:** Qian-Hao Zhu, Ming-Bo Wang

**Affiliations:** CSIRO Plant Industry, GPO Box 1600, Canberra ACT 2601, Australia; E-Mail: ming-bo.wang@csiro.au

**Keywords:** long non-coding RNA, RNA-seq, natural miRNA target mimic, chromatin modifier

## Abstract

The past decade has seen dramatic changes in our understanding of the scale and complexity of eukaryotic transcriptome owing to the discovery of diverse types of short and long non-protein-coding RNAs (ncRNAs). While short ncRNA-mediated gene regulation has been extensively studied and the mechanisms well understood, the function of long ncRNAs remains largely unexplored, especially in plants. Nevertheless, functional insights generated in recent studies with mammalian systems have indicated that long ncRNAs are key regulators of a variety of biological processes. They have been shown to act as transcriptional regulators and competing endogenous RNAs (ceRNAs), to serve as molecular cargos for protein re-localization and as modular scaffolds to recruit the assembly of multiple protein complexes for chromatin modifications. Some of these functions have been found to be conserved in plants. Here, we review our current understanding of long ncRNA functions in plants and discuss the challenges in functional characterization of plant long ncRNAs.

## 1. Introduction

Whole-genome tiling array and RNA sequencing (RNA-seq) have revealed that the transcription landscape in eukaryotes is much more complex than had been expected, with a high proportion of novel transcripts generated from intergenic regions and promoters of annotated genes [1]. Meanwhile, natural antisense transcripts, which are RNA molecules transcribed from the opposite DNA strand and overlapping in part or full with the sense transcripts [2], have been shown to be a pervasive feature of mammalian genomes [3,4]. Antisense transcripts were also found in ~30% of annotated genes in *Arabidopsis* [5]. Although ~90% of the human genome is transcribed [6], the ENCODE project demonstrated that only ~1.2% of the genome encodes proteins [7], suggesting that a large proportion of the eukaryotic genome produces RNA molecules that have no protein-coding capacity, namely non-coding RNAs (ncRNAs).

ncRNAs are arbitrarily grouped into short (<200 nt) and long ncRNAs (lncRNAs; >200 nt). The importance of short ncRNAs, including siRNAs, miRNAs and piRNAs, in transcriptional and posttranscriptional regulation of gene expression has been well recognized and the molecular mechanisms of short ncRNA-mediated regulation have been well understood [8,9]. In contrast, the regulatory roles of lncRNAs are only beginning to be recognized and the molecular basis of lncRNA-mediated gene regulation is still poorly understood. Studies on a small number of lncRNAs in animals have shown that they are involved in multiple levels of the gene regulation. These lncRNAs have been shown to mediate epigenetic changes through recruitment of the Polycomb repressive complex (PRC) [10,11,12], to act as decoy for splicing factors [13] and to compete for miRNA binding sites [14,15,16,17,18]. In comparison to animals, plants have fewer lncRNAs been identified [19,20] and functionally characterized [21,22,23,24]; however, the emerging picture is that the regulatory functions of plant lncRNAs are largely similar to animal lncRNAs. In this review we first provide a brief introduction of the methodologies used in lncRNA identification, and then summarize recent progresses in functional characterization of lncRNAs in plants. We also discuss the challenges in unveiling the functions of lncRNAs. Our focus in this review is on the lncRNA functions that are independent of siRNA-directed gene silencing pathways; functions of RNA polymerase V-dependent lncRNAs involved in RNA-directed DNA methylation and precursor transcripts of trans-acting siRNAs (tasiRNAs) will not be discussed. We refer readers to excellent recent reviews on these topics [25,26,27].

## 2. Discovery of lncRNAs

### 2.1. *In Silico* Identification

The rationale for *in silico* identification of lncRNAs is that they can be distinguished from protein-coding mRNAs based on the absence of discernible open reading frames (ORFs). The starting data for *in silico* identification can be sequences of cDNAs or Expressed Sequence Tags (ESTs) deposited in public databases or novel transcripts generated by full-length cDNA cloning, tiling arrays and RNA sequencing (see below). Usually cDNAs or EST sequences are first compared with genomic sequences to remove those overlapping with protein-coding genes; the remaining sequences are then subjected to ORF prediction. The threshold of ORF length is usually 70–100 amino acids, *i.e*., RNAs with a predicted ORF of <70–100 amino acids would be treated as lncRNAs. Existing ORF prediction programs include GeneMark.hmm [28], GenScan [29], ESTScan2 [30], ANGLE [31] and ORF-Predictor [32]. More sophisticated bioinformatics tools for estimating the protein-coding potential of a RNA sequence include CRITICA [33], DIANA-EST [34], CSTminer [35], CONC [36], Coding Potential Calculator [37], integrated ncRNA finder [38] and RNAcode [39]. The *in silico* approach has been successfully applied to identifying lncRNAs in both plants [19] and animals [31,40,41].

### 2.2. *De Novo* Identification

#### 2.2.1. Whole-Genome Tiling Array and RNA-seq Approaches

Full-length cDNA sequencing is the gold standard for determining exonic structure and coding or non-coding potential of a transcript; however, this approach is time-consuming and expensive. Furthermore, RNAs with low-level expression, a characteristic of most lncRNAs, would be hard to uncover using traditional cDNA cloning strategies. Tiling DNA microarray, designed for genome-wide high-resolution transcriptome analysis, provides an alternative for detection of lncRNAs and their expression. Using this approach, a large number of uniquely transcribed intergenic regions and stress-induced novel transcripts were found in rice [42] and *Arabidopsis* [43,44], respectively. These novel transcripts provided a rich source for lncRNA discovery. In addition, a single nucleotide resolution array designed for the *Arabidopsis FLC* (*FLOWERING LOCUS C*) locus and its 50-kb surrounding region uncovered a number of non-coding transcripts antisense to *FLC* [24]. However, tiling arrays rely on the existing knowledge of genome sequence. This technology allows for the identification of novel exons or transcriptional units but it does not provide information about their connections. Furthermore, it still suffers from a lack of high sensitivity in detecting rare transcripts due to high levels of background, cross-hybridization of related sequences and saturation of signals.

Some of the drawbacks with tiling arrays can be circumvented by RNA-seq, which has emerged as a new technology for tackling the complexity of eukaryotic transcriptomes in an unbiased manner [6,45,46]. RNA-seq is able to detect transcripts that are missing or incomplete in the reference genome and allows for accurate quantification of expression levels, making it an ideal approach for lncRNA discovery. With an ultra sequencing depth RNA-seq can be used to discover rare transcripts that are expressed in just a few cells within a tissue. For instance, by combining RNA-seq with targeted RNA capture, a ~4,607 fold coverage was achieved for the targeted human genomic regions. With this RNA-seq depth, rare alternative splicing variants of the lncRNA *HOTAIR* were identified, and lncRNAs expressed in only a small subpopulation of the cells sampled could be detected [47]. In another study, ~2,000 novel transcribed regions that do not link to any annotated gene models were identified by a comprehensive investigation of the *Drosophila melanogaster* transcriptome using tiling arrays in combination with RNA-seq*.* Approximately two thirds of these novel transcripts have an ORF less than 100 amino acids, including a multi-exon lncRNA in the well-studied Bithorax complex, which is expressed in embryos and adult males but not in females [48]. When combined with RNA immunoprecipitation, RNA-seq could facilitate the identification of lncRNAs associated with specific RNA-binding proteins and chromatin remodeling complexes [49,50].

#### 2.2.2. Chromatin Signature-Based Approach

An actively transcribed region is usually defined by a K4-K36 domain, *i.e*., an active promoter marked by H3K4me3 (trimethylation of lysine 4 of histone H3) in combination with a transcribed region marked by H3K36me3 (trimethylation of lysine 36 of histone H3). By searching K4-K36 domains in intergenic regions in the human and mouse genomes, a large number of lncRNAs, named as long intergenic ncRNAs (lincRNAs), were found in these two species. A significant number of these lincRNAs are conserved between human and mouse [49,51], suggesting that they are functional. The finding that ~38% of these lincRNAs were physically associated with chromatin modifying complexes, such as Polycomb repressive complex 2 (PRC2) and/or CoREST, further suggests a regulatory role of these lincRNAs [49]. This approach has not yet been adopted in plants mainly due to lack of genome-wide H3K36me3 data although genome-wide H3K4me3 landscape has been established in *Arabidopsis* [52].

## 3. Molecular Functions of lncRNAs in Plants

### 3.1. LncRNA as Natural miRNA Target Mimic

Phosphate is an essential macronutrient for plant growth and development. Plants must not only absorb considerable amounts of phosphate from the soil but must also have a sophisticated regulatory mechanism to maintain phosphate homeostasis throughout the plant to meet the growth and metabolic requirements of each tissue. miRNAs have been shown to be an essential component of this complex regulatory system [53,54,55,56,57]. miR399, which is expressed in companion cells and phloem, is strongly induced by phosphate starvation [53]. Consequently the expression level of *PHO2*, a target of miR399 and encoding an E2 ubiquintin conjugase-related enzyme (UBC24), is repressed due to miR399-mediated mRNA cleavage [53,54,55,56]. Low *PHO2* activity leads to enhanced expression levels of two root-specific phosphate transporter genes, *Pht1;8* and *Pht1;9* [53,54], resulting in increased phosphate uptake. Besides miR399, *Induced by Phosphate Starvation1* (*IPS1*), a member of the *TPS1*/*Mt4* gene family that was first identified in tomato and *Medicago truncatula* [58,59] and then in other plant species including rice [60] and *Arabidopsis* [61,62], is also induced by phosphate starvation. *IPS1* does not encode a protein, and only a 23-nt long sequence motif is conserved among the members from different plant species [21,63,64]. This 23-nt motif is partially complementary to miR399 with a 3-nt central mismatch corresponding to positions 11–13 of miR399. As miRNA-mediated RNA cleavage usually occurs between nucleotides 10 and 11 relative to the 5' end of the miRNA, this central mismatch disrupts crucial base-pairing between miR399 and *IPS1* and hence inhibits miR399-mediated cleavage of *IPS1*. This observation leads to the hypothesis that *IPS1* functions as a non-cleavable target mimic of miR399 to sequester miR399 which in turn attenuates miR399-mediated repression of *PHO2* [21]. Indeed, transgenic plants overexpressing *IPS1* increased the transcript and protein levels of *PHO2*, whereas transgenic plants overexpressing a cleavable *IPS1* did not [21]. Thus, the increased expression of *IPS1* under phosphate starvation appears to counter-balance the effect of increased miR399 accumulation under the same condition, resulting in fine tuning of *PHO2* expression and phosphate uptake [63].

Such inhibition of miRNA activity by an endogenous noncleavable ncRNA target has been termed as target mimicry [21]. Recent discovery of competing endogenous RNAs (ceRNAs) in animal and human cells indicates that target mimicry may be a widespread phenomenon, where non-coding and coding RNAs with similar miRNA target sites could affect each other’s activity.

The first example of ceRNA is the human pseudogene *PTENP1*, which is related to the tumor suppressor gene *PTEN* and produces a naturally occurring ncRNA. Both *PTEN* and *PTENP1* contain many conserved miRNA binding sites in their 3' untranslated regions (UTRs). *PTENP1* was found to regulate the expression of *PTEN* by acting as a decoy for miRNAs that bind to the common sites in the 3' UTRs of *PTENP1* and *PTEN* [14,16,18]. More recently, a muscle-specific lncRNA, *linc-MD1*, has been shown to regulate the expression of *MAML1* and *MEF2C* by sequestration of miR-133 and miR-135 that target the two genes. *MAML1* and *MEF2C* are two transcription factors that activate muscle-specific gene expression, controlling the timing of muscle differentiation. Consistently, downregulation or overexpression of *linc-MD1* resulted in a decreased or increased accumulation of myogenic marker genes in mouse myoblasts, which leads to retardation or acceleration of the muscle differentiation program, respectively [15]. Apart from these individual examples, transcripts of ~7,000 genes have been shown to potentially act as natural miRNA target mimics to regulate the establishment of oncogenic pathways in glioblastoma in human [17]. These results suggest that target mimicry or ceRNA network plays an important role in cell differentiation and tumorigenesis [17,65].

Besides its biological significance, target mimicry has provided an alternative approach for functional characterization of miRNAs. In plants, characterization of gene function has relied largely on the use of genetic knockout mutants caused by T-DNA or transposon insertion. However, because of the small size of *MIRNA* genes and the existence of multiple, highly conserved members in most plant miRNA families, it has been extremely laborious and time-consuming to obtain a corresponding null mutant plant line of a *MIRNA* gene [66]. Target mimicry has therefore been exploited as an alternative approach for functional characterization of miRNAs. The usefulness of this approach has been demonstrated by the closely resembled phenotypes observed in plants transformed with target mimicry constructs and in plants either overexpressing miRNA-resistant targets or harbouring a T-DNA insertion in *MIRNA* genes [21,67]. In animals and human, artificial miRNA sponge, a strategy similar to target mimicry in plants, has been widely used in characterization of miRNA functions [68,69]. In addition, artificial miRNA sponge has also been investigated for potential therapeutic applications in human diseases such as cancer and cardiac disorders associated with miRNA misregulation.

### 3.2. LncRNA Guiding Recruitment of Chromatin Modifiers

Studies in animals and plants have demonstrated that chromatin modifications are important for tissue-specific gene expression and for genome reprogramming during development [70,71]. Chromatin modifications at a certain locus are believed to be initiated by site-specific recruitment of chromatin modifying complexes. Several lncRNAs, such as *Air*, *HOTAIR*, *Xist* and *Kcnq1ot1*, have been shown to target repressive histone-modifying activities and direct epigenetic silencing through a molecular interaction with specific chromatin domains in animals and human [12,72,73,74,75,76,77]. In addition, hundreds of lncRNAs have been shown to co-purify with various components of chromatin modifying complexes in co-immunoprecipitation assays in human [49]. In plants, lncRNA-mediated chromatin modification has so far only been demonstrated in the *FLC* locus in *Arabidopsis* [23].

*FLC* acts as a floral repressor that confers a requirement for vernalization, a process by which certain plants acquire competence to flowering in spring by sensing prolonged exposure to winter cold [78,79]. Molecular studies have shown that both activation and repression chromatin remodelling complexes are involved in the regulation of *FLC* expression [80]. Vernalization induces a Plant HomeoDomain (PHD) finger containing protein, VERNALIZATION INSENSITIVE 3 (VIN3), and promotes association of VIN3 with PRC2 to stably repress the expression of *FLC* [81,82] through PRC2-mediated deposition of H3K27me3 marks at the *FLC* locus. The level of PRC2 occupancy at *FLC* is correlated with the level of H3K27me3 and consequently the degree of repression of *FLC* [81,82]. Increased occupancy of PRC2 followed by increased level of H3K27me3 at the *FLC* chromatin is necessary for the stable maintenance of vernalization-induced *FLC* repression. PRC2 is a conserved repressive chromatin modifier [83]. In human, *HOTAIR*, an lncRNA generated from the *HOXC* locus, has been shown to mediate epigenetic changes at the *HOXD* locus *in trans* by recruiting PRC2 [12]. Further studies indicate that interaction between lncRNAs and chromatin modifying complex seems to be a general mechanism for epigenetic silencing in animals [84]. These findings encouraged plant scientists to investigate if lncRNAs are generated from the *FLC* locus and if they play a role in the repression of *FLC* expression.

Two classes of lncRNAs are identified from the *FLC* locus. The first class is *COOLAIR*, including long and short versions of lncRNAs that are transcribed in antisense orientation relative to *FLC* by a promoter located downstream of *FLC*. The expression levels of *COOLAIR* increase during vernalization, and induction of *COOLAIR* by vernalization coincides with a reduction of *FLC* but is earlier than the onset of other vernalization makers, such as *VIN3* [24]. This observation led to the suggestion that *COOLAIR* is involved in early, cold-dependent transcriptional silencing of *FLC* [24]. The nature of antisense orientation between *COOLAIR* and *FLC* and that the long version of *COOLAIR* transcripts extend beyond the transcriptional start site of *FLC* suggests a possible role of *COOLAIR* through transcriptional interference [24]. However, a more recent study, using multiple T-DNA insertion lines across the *FLC* and *COOLAIR*, showed that the transcription of *COOLAIR* is not required for the initial repression of *FLC*; instead the promoter and the first exon of the *FLC* gene are sufficient to initiate *FLC* repression during vernalization [85]. In addition, *COOLAIR* does not physically interact with PRC2 [23].

The second class of lncRNAs, *COLDAIR* that was uncovered by tiling RT-PCR, are transcribed from the first intron of *FLC* in the same direction as *FLC* [23]. Similar to *COOLAIR*, *COLDAIR* is also transiently induced by vernalization, but its peak expression time point is observed later than that of *COOLAIR*. The *COLDAIR* transcript interacts directly with CURLY LEAF (CLF), one of the components of PRC2, and can be co-purified with PRC2, indicating a direct role of *COLDAIR* in the recruitment of PRC2 to *FLC*. Recruitment and deposition of PRC2 at *FLC* increase the level of H3K27me3 at *FLC* chromatin after vernalization [23]. Knockdown of *COLDAIR* using RNAi compromises cold-mediated H3K27me3 enrichment and the vernalization response. In addition, the vernalization-induced repression of *FLC* is not maintained once plants return to warm conditions in the *COLDAIR* knockdown lines. These results together with the observation that the repression of *FLC* cannot be maintained in PRC2 component mutants suggest that *COLDAIR* is required for establishment and maintenance of the stable silencing state of *FLC* [23,86]. These results also suggest that lncRNA-mediated recruitment of PRC2 and gene repression is an evolutionally conserved mechanism in eukaryotes [23].

A growing body of evidence supports the notion that lncRNAs are key regulators of chromatin state through interacting and recruiting chromatin remodelling complexes to specific genomic loci. Several models, by which lncRNAs tether or guide chromatin modifying complexes to their specific destinations, have been proposed [12,84,87]. Meanwhile, genome-wide approaches for isolation of lncRNAs associated with chromatin or chromatin modifiers [50,88] and for identification of lncRNA occupancy [89] have been established. However, the nature and sites of lncRNA-chromatin interaction are still largely unknown and more studies are required to uncover the exact mechanism(s) controlling the interaction between lncRNAs and chromatin modifying complexes.

### 3.3. LncRNA as Molecular Cargo for Protein Re-Localization

The early nodulin gene *Enod40*, first identified in soybean and *Medicago sativa* ssp. *varia* [90,91], is a plant gene that participates in the regulation of symbiotic interaction between leguminous plants and soil bacteria [91,92]. *Enod40* is rapidly induced by rhizobia in the root pericycle and in the dividing cortical cells of the nodule primordium during the symbiotic interaction [93]. Transgenic approach confirmed a role of *Enod40* in nodulation [94]. *Enod40* is highly conserved among legumes and is also present in various non-legume species, such as rice [95,96]. The *Enod40* transcript lacks long open reading frames, but encodes two short peptides (12 and 24 amino acid residues in soybean; and 13 and 27 amino acid residues in *M. truncatula*) [97,98]. Translation of these two short peptides is directly related to the biological activity of *Enod40* in *M. truncatula* [98]. In Soybean, these peptides were shown to bind specifically to sucrose synthase, suggesting a role of *Enod40* in the regulation of sucrose utilization in nodules [97]. However, two features of the *Enod40* transcript suggest that the general mechanism of action of *Enod40* may be achieved through its RNA molecule rather than the short peptides. Firstly, the *Enod40* RNA is highly structured and contains a highly stable RNA secondary structure. Analysis of *Enod40* transcripts from numerous leguminous species revealed five conserved domains [99] and at least two domains are absolutely conserved in all currently found *Enod40* homologues [95]. Secondly, one of the two short peptides is not always conserved and the highest conservation at the nucleotide level is observed in the region outside the conserved peptides [95]. In addition, the overall configuration of the secondary structure elements in the *Enod40* RNA is more conserved than the ORFs encoding short peptides [95].

The importance of the secondary structure of *Enod40* was demonstrated in *M. truncatula*. Plants transformed with an altered *Enod40*, in which the RNA structural elements were deleted while the proper translation of short peptides was retained, decrease its role in stimulation of cortical cell division and formation of nodules [98]. More importantly, *Enod40* has been shown to directly interact with MtRBP1 (*Medicago truncatula* RNA binding protein 1), a constitutively expressed RNA-binding protein identified by yeast three-hybrid screening, and play a role in re-localization of MtRBP1 from nuclear speckles into cytoplasmic granules during nodulation in *M. truncatula* [22]. This re-localization of MtRBP1 was only observed in *Enod40*-expressing plant cells and was not affected by impaired activity of peptide translation [22], suggesting that the *Enod40* RNA rather than the *Enod40*-encoded short peptides is important for the MtRBP1 re-localization. This study demonstrated that *Enod40*, like *Mei2p* in the fission yeast, is part of the nucleocytoplasmic trafficking machinery [100].

Recently, two small nodulin acidic RNA-binding proteins, MtSNARP1 (*Medicago truncatula* small nodulin acidic RNA-binding protein 1) and MtSNARP2, were also identified to interact with *Enod40* in *M. truncatula* [101]. However, the RNA-binding activity of MtSNARP2 does not seem to be sequence specific because MtSNARP2 is able to bind the entire *Enod40* RNA and synthetic RNA oligos as well. In addition, the exact binding sites in *Enod40* RNA have not yet been determined although the 5' and 3' regions of the *Enod40* transcripts are important for its interaction with MtRBP1 [22].

## 4. Challenges for Decoding the Functions of lncRNAs

RNA-seq technology provides a powerful tool for unbiased profiling of transcriptomes in complex organisms; however, it is still a long way from reaching the limit of the transcriptome as most lncRNAs are very lowly expressed [47]. Development of new methodologies, such as RNA CaptureSeq [47] and single-cell transcriptome profiling [102], has greatly increased the chance to identify rare transcriptional events taking place in specific genomic regions or cell types of interest. The ongoing development of the direct RNA sequencing technology, in which single RNA molecule is sequenced directly without prior conversion to cDNA, promises reduction of artifacts associated with the current RNA-seq technology during cDNA synthesis [103]. However, further technical innovation and revolution is required to routinely identify rare transcripts in a genome-wide scale in animals and particularly in plants.

Currently, distinguishing lncRNAs from protein-coding mRNAs depends solely on ORF prediction. However, several points need to be considered. First, the accuracy of ORF prediction relies on the completeness of the full-length status of the input sequences. Second, some transcripts such as *Enod40* lack long ORF but do contain short ORF(s) that has potential to encode short peptides [97,98]. For such transcripts, assigning a function to the RNA molecule or to the short ORF(s) is not a trivial task. While evolutionary conservation of amino acid sequences can be used to assess the functionality of short ORFs, and information on the intracellular localization of a RNA transcript also offers clues for its functionality, the ultimate solution should be to assess the activity of the RNA molecule or its encoded short peptide(s) using biochemical approaches [104]. Third, some protein coding RNAs could have additional structural functions that are unrelated to their translation product, as demonstrated by the identification of a class of coding transcripts with a role through their RNA molecules in maintaining a decondensed and biologically active interphase chromatin conformation in human and mouses [105]. Thus, when it comes to the functional characterization of single transcripts, the presence of an ORF does not necessarily exclude the existence of additional regulatory functions at the RNA level; and *vice versa* [106].

Despite a number of lncRNAs have been demonstrated to function in guiding chromatin modifying complexes to specific genomic loci, the exact nature of the chromatin binding sites is yet to be determined. A new technique, Chromatin Isolation by RNA Purification (ChIRP)-seq, is suitable for genome-wide characterization of chromatin-binding sites [89]. Applying this technique to three lncRNAs has revealed that lncRNA binding sites resemble transcription factor binding sites in being focal, numerous and sequence-specific [89]. Future works are required to elucidate how a single stranded RNA molecule interacts with a specific double-stranded genomic region.

## 5. Conclusions

A large number of lncRNAs have been identified in animals. Sequence conservation and tissue-specific expression patterns strongly suggest that these lncRNAs are more than just transcriptional noise. With the increased cases of confirmed functional lncRNAs, it is becoming increasingly evident that lncRNAs play important roles in diverse cellular processes. However, for the majority of lncRNAs their biological functions remain unknown, and their precise mechanisms of action are yet to be determined. In plants, a number of lncRNAs have also been identified by *in silico* or *de novo* approaches. While functional characterization of plant lncRNAs is still in its infancy, studies so far suggest that they function via similar mechanisms to animal lncRNAs. It can be anticipated that a diverse array of new molecular functions will emerge for plant lncRNAs with increased numbers of new plant lncRNAs being identified and characterized.
